# The dynamics of negative stereotypes as revealed by tweeting behavior in the aftermath of the Charlie Hebdo terrorist attack

**DOI:** 10.1016/j.heliyon.2020.e04311

**Published:** 2020-08-06

**Authors:** Yousri Marzouki, Eliza Barach, Vidhushini Srinivasan, Samira Shaikh, Laurie Beth Feldman

**Affiliations:** a Department of Social Sciences, Qatar University, Qatar; b University at Albany, State University of New York, Albany, NY, USA; c University of North Carolina at Charlotte, Charlotte, NC, USA; d Haskins Laboratories, New Haven, CT, USA

**Keywords:** Psychology, Stereotypes, Tweeting behaviour, Social network analysis, Sentiment analysis, Islamophobia

## Abstract

We describe the evolution of a stereotype as it emerged in tweets about the Charlie Hebdo terrorist attack in Paris in early 2015. Our focus is on terms associated with the Muslim community and the Islamic world. The data (400k tweets) were collected via Twitter streaming API and consisted of tweets that contained at least one of 16 hashtags associated with the Charlie Hebdo attack (e.g., #JeSuisCharlie, #IAmCharlie, #ParisAttacks), collected between January 14th and February 9th. From these data, we generated pairwise co-occurrence frequencies between key words such as “Islam”, “Muslim(s)”, “Arab(s)”, and “The Prophet” and possible associates such as: “terrorism”, “terror”, “terrorist(s)”, “kill(ed)”, “free”, “freedom” and “love”. We use changes in frequency of co-occurring words to define ways in which acute negative and positive stereotypes towards Muslims and Islam arise and evolve in three phases during the period of interest. We identify a positively-valenced backlash in a subset of tweets associated with the “origins of Islam”. Results depict the emergence and transformation of implicit online stereotypes related to Islam from naturally occurring social media data and how pro-as well as anti-Islam online small-world networks evolve in response to a terrorist attack.

## 1 Introduction

There is a growing appreciation that social media platforms can reveal the attitudes and emotional reactions of users. Referred to as *mining naturally occurring data*, the associated methods are considered by some researchers to constitute a paradigm shift in defining psychological data because they differentiate between data derived from traditional lab-based experiments and from computational modeling (Goldstone and Lupyan, 2016). Analyses with many applications have proven efficient to gauge the collective “temperature” of people's opinions from “likes” and “dislikes” in marketing (e.g., Andrew and Toubia, 2010; Fischer and Reuber, 2011), public health (Paul and Dredze, 2011), and politics (e.g., Browne et al., 2015; Marzouki et al., 2012; Ruz et al., 2020). Several paramount examples are the 2008 Obama campaign, the 2011 Arab revolution(s) and Brexit. The first campaign of Obama relied heavily on social media to promote his candidacy to a new subset of the population with the result that Obama was among the top five most popular worldwide personalities on Twitter (Boulianne, 2015; Carr, 2008). During the 2010 Tunisian revolution, massive interaction through Facebook by a large proportion of the Tunisian youth when the country underwent an unprecedented media blackout was a key element in the overthrow of its dictatorship (Marzouki et al., 2012). In addition, Del Vicario et al. (2017) explored news portrayal of Brexit in the UK on Facebook and emphasized the impact of selective exposure and confirmation bias on the direction of spread of information. In particular, their analysis of the evolution of core narratives in online discussions showed the presence of highly polarized groups that exhibited significant differences in terms of emotional responses (e.g., likes and dislikes) in comments related to the Brexit-related topics. More recently, Tavazoee et al. (2019) showed that Trump tweeting style and consistency has increased his popularity in social media.

Less explicit than counts of likes and dislikes to study sentiment, data based on affective dimensions such as ratings of the positive versus negative valence of key words in social media posts provide reliable indicators that shed light on reactions and attitudes of its users (e.g., Warriner et al., 2013 for details). Further, valence for unrated words can be predicted from ratings of the words with which it co-occurs (Recchia and Louwerse, 2015). Purportedly, ratings for affective dimensions tend to be stable across cultural groups (Riegel et al., 2017) although arousal is allegedly less universal than valence (Lim, 2016). Finally, the reliability of word ratings of valence and other affective dimensions of sentiment exist in multiple languages including Italian (Recchia and Louwerse, 2015), Spanish (Stadthagen-González et al., 2018) and Chinese (Lin and Yao, 2016) in addition to English. Therefore, many prefer to rely on affective ratings of words as compared to surveys and questionnaires to understand emotion because of possible demand characteristics of the interview process such as how questions are posed. When words are analyzed in terms of affective measures based on ratings for individual words, social media platforms like Twitter become a reliable indicator of the candid sentiment and uncensored attitudes of its millions of users (Marr, 2016). There are many measures that have been applied. For example, centrality analyses in social networks within an active Twitter community capture the structure of a small-world network (e.g., the word usage patterns and lexical diversity) whilst interacting locally with a bordering community. The “single-mindedness” of the arguments exchanged within the network maintains the cohesiveness and boundary of the community (Bodrunova et al., 2018; Ch'ng, 2015).

Twitter data have many other features that contribute to its special value and potential informativeness that depends on patterns of language usage. From a methodological perspective, online data are constrained because the length of a tweet cannot exceed a specified number of characters (the limit was 140 characters during the Charlie Hebdo events and became 280 characters in 2017). This constraint is related to another characteristic that makes Twitter data especially informative about language and cognition. More specifically, the length limit of a tweet coerces users to reduce the essence of their message to a limited number of characters, words, and emojis. A second relevant characteristic derives from the spontaneity and the inherent temporal dynamics of Twitter as attested by timestamps that serve to preserve an accurate record so that it is possible to keep track over time of the distributional behavior of tweets either collectively or as single retweets. For example, Fusaroli et al. (2015) identified distinct time scales in online human communication each of which was marked by the presence of interactional dynamics of the unfolding discussions related to massively shared experience of a political event. Thus, time scales differ with respect to the observed behavior: short-term conversational dynamics, mid-term content, and longer-term attentional entrainment as discernable from large-scale attention bursts and decays. Fusaroli et al. (2015) detected these dynamics in online behaviors by analyzing users’ complex interactions in the context of a major political event.

In summary, it is possible to track who uses or reuses a particular tweet and when, relative to other tweets in a corpus. However, the level of interaction can reach exponential complexity given the fact that numerous tweets arrive in a very short time span thus making it impossible to trace back a single chain of influence among them. Because of these complex patterns of connectivity, it is usually pointless to determine a single direction of influence and finally to designate who is “guiding” the conversation.

### 1.1 Origins of the twitter corpus

On the 7^th^ of January 2015, Islamist gunmen armed with assault rifles entered the office of the satirical newspaper *Charlie Hebdo* in Paris and opened fire on the publication's journalists and cartoonists. The editor, Charb, along with seven other members of the Charlie Hebdo staff were killed. This terrorist attack became known as the Charlie Hebdo attack and has attracted worldwide discussion along the theme of freedom of expression in media outlets. Arunima and Ruchi (2020) showed that the Paris attack even received far more coverage from both CNN and Al Jazeera compared to Beirut attack that happened the same year. In social media, the theme of freedom of expression has taken various forms including Twitter's hashtag #JeSuisCharlie. Several seminal studies have examined Twitter data to understand people's reactions towards the Charlie Hebdo attack. Giglietto and Lee (2015) analyzed the co-occurrence patterns of words with accompanying hashtags and found that tweets took on various characteristics of the users' online discussions and reactions. The analysis of the content of tweets with the hashtag #JeNeSuisPasCharlie revealed systematic hidden patterns around specific keywords such as “activism”, “grief”, “resistance”, “ethnocentrism” and “Islamophobia” in the 74,074 posted tweets collected between 7th and 11th of January 2015 (see also, Giglietto and Lee, 2017). Shaikh, Feldman, Barach, and Marzouki (2016) examined how the affective valence and concreteness of English words in tweets from the Charlie Hebdo terrorist attack varied with pronoun use and rate of participation. After extracting the valence score from an existing valence ratings lexicon based on 14,000 words (Warriner et al., 2013), Shaikh et al. (2016) found that tweets with pronouns tended to be more negatively valenced than those without and trended slightly *more* negative over time. Interestingly, an analysis of tweet affect in the sub-community of supertweeters (users with the highest rate of tweet generation) got *less* negative over time. In a recent related study that focused on the francophone “Twittersphere”, Bodrunova et al. (2018) reported that the negativity measured with an automated detection algorithm in the users' discussions was associated more strongly with #jenesuispascharie than with #jesuischarlie especially among non-institutional users (government institution, associations, ministries, etc.) people. By comparison, institutional users exhibited more neutral and positive sentiment in their tweets. They also reported that the increase in supertweets about truth and the decrease in supertweets about insult/offense and hypocrisy appeared to be capturing a backlash phenomenon. The latter could be interpreted as an idiosyncratic small-world network that retains boundaries and thus the cohesiveness of the community by maintaining particular content in its arguments (Ch'ng, 2015). We can hypothesize that such an idiosyncratic network (the supertweets in our corpus) may reflect an analogous psychosocial appraisal among individuals who violate the predominant negative stereotype - Islamophobia and biases towards Muslims and Islam per se- (e.g., Rudman and Fairchild, 2004; Rudman and Phelan, 2008).

In general, a stereotype reflects individuals' cognitive structures that pertain to knowledge, beliefs, and expectancies about another's social group (Hamilton and Trolier, 1986). Stereotypes are considered as part of the cognitive component of prejudice also referred to as hostile or negative attitude towards people in a distinguishable group (Aronson et al., 2018). The two other components of prejudice are affective and behavioral. Stereotypes is a mental pigeonholing process that puts people in different categories based on certain characteristics. The functional role of stereotypes is believed to be an adaptive mechanism of our brain in order to make sense of our physical and social worlds (Cikara and Van Bavel, 2014).

Islamophobia and xenophobia towards Muslims captures those structures in the stereotype towards Muslims (e.g., Allen, 2007). In the particular case of Charlie Hebdo, the Muslim community was targeted monolithically as the culprit behind any and all terrorist activity in what is referred to as the post 9–11 Islamophobia (e.g., O'Connor, 2016). From consideration of relevant events and the scope of the international reactions to them, it may be observed that Islamophobia was on the rise by the time of the event. One example of this change in attitudes and beliefs was the strong anti-Islam reactions after the deadly attacks on Charlie Hebdo (Ebbitt, 2015). Giglietto and Lee (2015) analyzed 74,047 tweets in the context of Islamophobia. They coded differently in their corpus the tweets containing #JeSuisCharlie vs. the tweets containing #JeNeSuisPasCharlie. They noted after running cluster analyses on the most frequent words that the evolution of #JeNeSuisPasCharlie followed three phases where the phase 1 was called “Grief” about what happened, phase 2 was called “Resistance” where users exhibited some reservations, and the last one was called “Alternatives” by offering alternative frames for Charlie Hebdo such as hate speech and Euro-centrism. On the other hand, the analysis of Shaikh et al. (2016) is intriguing in that they reported a *decrease* over time in the diversity of negatively valenced words that co-occurred with words associated with the Muslim world for a well-defined subset of their tweeter based on the highest tweet contribution rates. In the present study, we seek to identify the source of this distinctive collective network embedded within the broader network of Twitter. Insights into implicit mechanisms of stereotype creation derive from tracking variation in the words that co-occur with key words associated with Islam.

### 1.2 The aim of the study

Although a long-lasting tradition of social theories has assumed that “key individuals systematically direct any collective action”, an analysis of interactions in an online environment suggests that the key individuals assumption for collective action no longer may be viable (e.g., Ross, 2011). Unhampered by the exigencies of face-to-face communication, old technologies and the material world, collective actions can arise spontaneously, remotely and without a leader (Marzouki et al., 2012; Ross, 2011). At the core of many mechanisms (e.g., cascading, spreading, and synchronizing) within the framework of complex social networks is the interaction among a limited set of elements, sometimes referred to as *influential nodes* (e.g., Chen & al., 2012). In social terms, the role of a single publicly recognized leader is supplanted by the idea of several influential individuals who may contribute along different dimensions and at different time scales so as to alter the actions and movements of the network. The framework within which we treat our data is grounded in complex dynamical systems where highly complex online behavior such as tweeting can emerge from very simple rules initiated by each agent (i.e., user) with a tremendous collective outcome as a result of a non-linear interaction between users in the network (Richardson et al., 2014).

In this regard, advances in social network analysis (SNA) allow one to measure and to track social influences that typically can occur when individuals adjust their behaviors and opinions according to the behaviors and opinions of others within a given network (e.g., Fusaroli et al., 2015; Leenders, 2002). These advances also allow the study of the personality of people at the scale of 'big data' (Boyd and Pennebaker, 2017). In the same vein, previous studies have shown that many language features including word count, pronoun patterns, and verb tense were good predictors of the network's cohesiveness and social dynamics (Gonzales et al., 2009). In addition, the study of emotion as it can be a significant vector of rapid social contagion for moral ideas and opinions in large-scale naturally occurring social networks on Twitter as shown by Brady et al. (2017). Brady et al. (2017) showed that the presence of moral-emotional words, which are often associated with evaluations of societal norms in response to perceived injustices committed in the world, increased the transmission of messages by approximately 20% per word.

In regards with emotions, Young-Il and Dongyoung (2016) analyzed the European Social Survey (ESS) and showed that the Charlie Hebdo attack affected negatively French respondents' wellbeing and more particularly the immigrants. In the same vein, Ring et al. (2018) study has revealed that people's adjustment disorders symptoms in the aftermath of a terrorist attack is significantly predicted by the salience of mortality in the news and media. In addition, Silva (2018) showed that public opinion's and political attitudes in France did not significantly changed in reaction to the 2015 Paris attacks. The author concluded that a significant change only depends on contextual vulnerability in countries where issues of immigrants and immigration is socially not stabilized.

In the present work, we highlight, unlike the traditional view that promotes the presence of leadership in collective behaviors, implicit processes through tweeting behaviors as distinguished from deliberate behaviors and directives. In order to further probe the dynamics of a network based on tweets from the Charlie Hebdo events, we have revisited the same corpus generated by Shaikh et al. (2016) with the following working hypothesis: The co-occurrence patterns between key words, as distinguished from individual word frequencies within the corpus as a whole, provide a novel and reliable perspective on implicit stereotype formation. To anticipate, we have identified a backlash effect whereby the overall initial anti-Muslim attitudes become progressively attenuated and superseded by a constellation of more positive attitudes towards Muslims. We hypothesize that this reversal is likely to have emerged from a core group of “network influencers” specifically; super-tweeters within the network who participate at higher rates and increase their rates of tweet generation as time passes. To establish whether and when the backlash in affect emerges, we examine the context in which key words associated with Islam occur. We hypothesize that Islam associated keywords (Islam, Muslim, Prophet, Isis) will co-occur in a context of words whose valence shifts to positive from negative. We identify this shift in valence as a *backlash effect* and track its emergence.

## 2 Data collection

The data we collected consisted of all the tweets that contained at least one of 16 hashtags pertaining to the Charlie Hebdo attacks collected via Twitter streaming API. The data were collected in March 2015 and the language of the tweets is English. The geolocation of tweets is not available for the majority of the IDs, which makes their origin unknown most of the time. Some studies have estimated that between 9% and 15% of active Twitter accounts are bots (Onur et al., 2017), yet, we were not able to detect algorithmically in our collection whether a tweet is originated from humans or bots. The final dataset consisted of 404,918 tweets from about 190,000 unique twitter ids that were shared between January 14th and February 9th 2015. The unique hashtags across the corpus are listed in Table 1.

**Table 1:** List of unique hashtags in our tweet collection.

#JeSuisCharlie	#IamCharlie	#MarcheRepublicaine	#JeSuisAhmed
#ParisShooting	#NousSommesCharlie	#LaFranceEstCharlie	#jesuisfranck
#lemondeestcharlie	#ThanksTheWorldFromFrance	#jenesuispascharlie	#parisattacks
#parisattacked	#parisattack	#ParisEstCharlie	#CharlieHebdo

## 3 Results

### 3.1 Valence of words that co-occur with keywords in the full data set (*N* = 55954)

As stated above, the context in which key words occur can provide a marker of attitudes and a dramatic shift in affective valence would signal a backlash effect that emerges over time in the network as a whole. Our focus on the weeks of February 2015, more than three weeks after the attack, covers an arguably but expectedly critical period for perception change. Indeed, the first reactions after the attack were marked by an emotional tone as conveyed by numerous media and social media outlets (e.g., Vasilopoulos et al., 2017). It is important in our study to track attitude change over time and how emotions drive users’ online responses.

**Figure 1:**
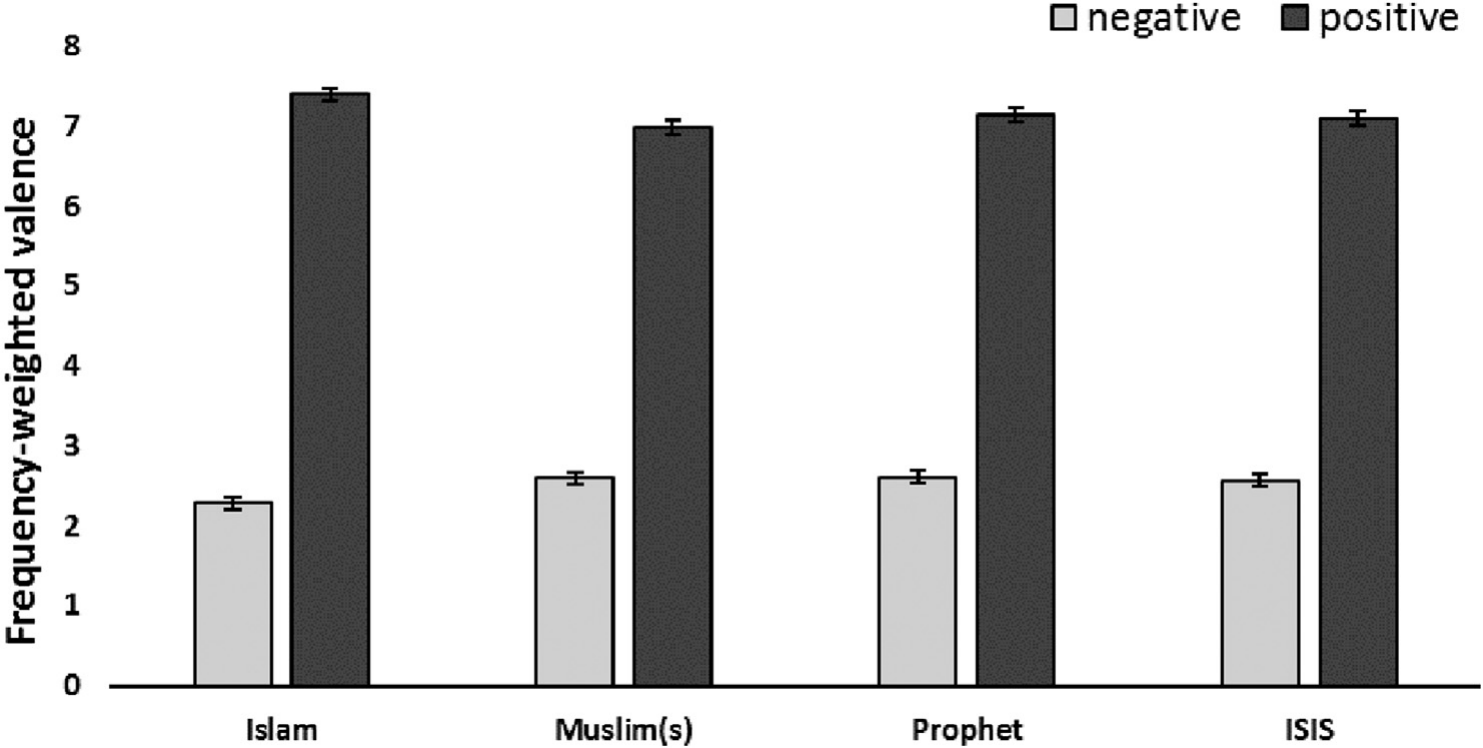
Frequency weighted valence scores for words that co-occur with one of the keywords Islam, Muslim(s), Prophet, and ISIS.

We selected Key words according to how frequently they appeared with the relevant hashtags (see Table 1 above). All key words referred to ideas central to Islam and the Muslim(s) people who practice it. We also add the word “Prophet” (including all its variants such as Muhammed). In addition, we included the prominent negative association that results in confusion and stereotypes, specifically ISIS. Valence values were extracted from an existing valence ratings lexicon (Warriner et al., 2013). We adopt a *weighted valence* measure that considers both the number of times each word occurs for those words that are strongly valenced (most extreme 25% positive, most extreme 25% negative) according to the Warriner et al. (2013) ratings. More specifically, the mean valence score is based on how often an extremely negatively (or positively) valenced word appears in context of all tweets that include the term “Islam”. Whether a tweet included a negation word was not considered. The total number of co-occurring words that are positively valenced is larger than for those that are negative (*N-positive* = 4608 vs. *N-negative* = 2581).Figure 1 illustrates the weighted valence of words in tweets that co-occur with the key terms: Islam, Muslim, Prophet and ISIS. Perhaps surprisingly, results show that all keywords were more likely to occur in tweets whose content is highly positive than in tweets whose content is negative.

**Figure 2:**
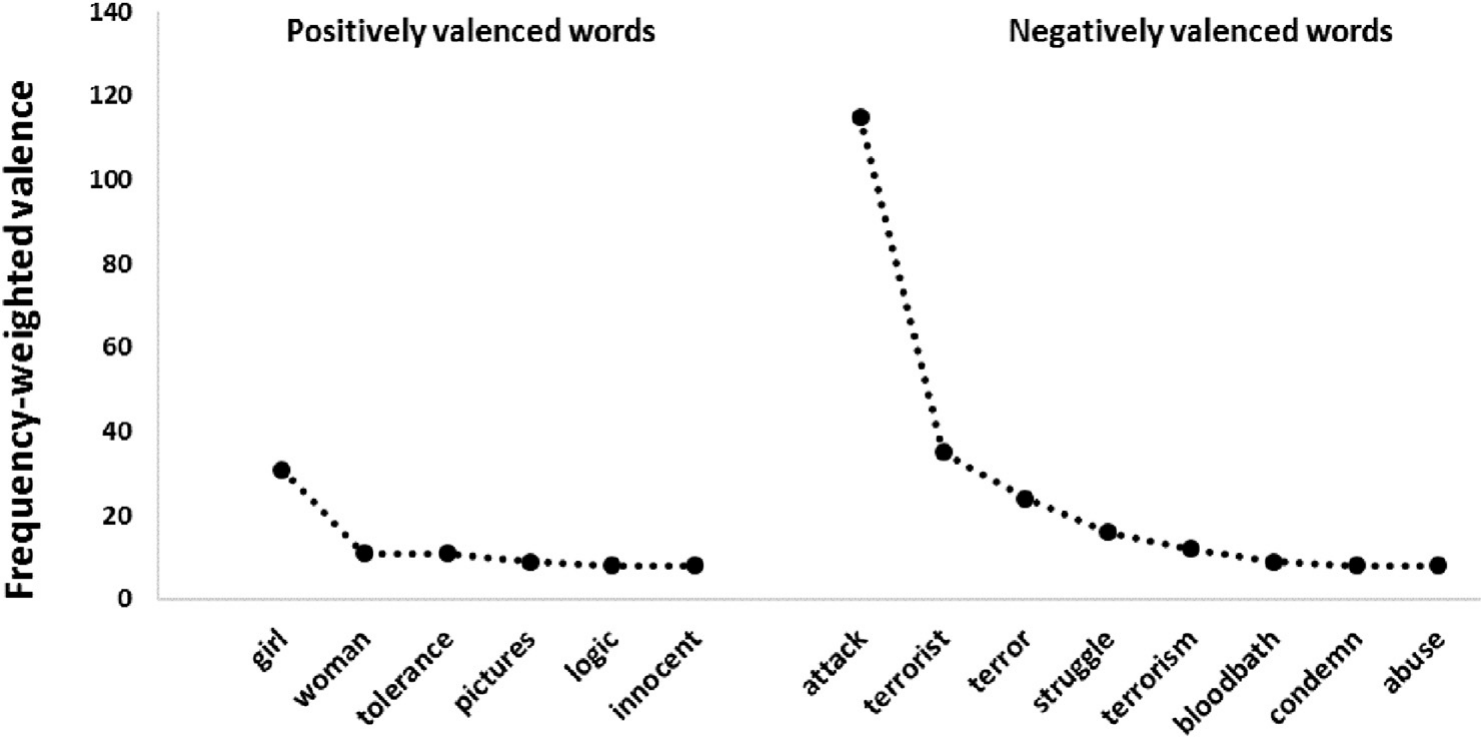
Strongly positively and negatively valenced words that co-occur with “Muslim” in tweets from February 2015.

The keyword “Muslim(s)” refers not only to the community of Islam believers but also to an adjective associated with the religion itself. Because of its complex senses, we have delineated in Figure 2, the most positively and the most negatively valenced words that co-occur with “Muslim(s)” during February 2015 in order to better understand its potential semantic ambiguity.

### 3.2 Lexical diversity of strongly valenced words

In a most general sense, the way the information is encoded, distributed within a network, regardless of its size, has been adapted by physicists and mathematicians to describe complex systems (Kleinberg, 2000; Newman, 2003). Since Rapaport's early attempts to model a friendship network (Rapaport, 1957), social network have been considered as the outcome of a stochastic process that has random and biased elements. The complexity of network's emerging patterns can be linked to the manner in which random and biased elements react and interact in local events (Skvoretz, 2002). Hence, measurements of entropy in social networks provide a novel way to capture the dynamic and adaptive behavior of humans (Zhao et al., 2011). In a similar way, measures from information theory can be applied to the frequency distribution of words and other linguistic structures as they appear in communication (Moscoso del Prado, Kostić and Baayen, 2004). Analyses and text mining techniques for large corpora of words often apply information theory measures to properties of those words such as their affective dimensions through three components: *valence* (the pleasantness of a stimulus), *arousal* (the intensity of emotion provoked by a stimulus), and *dominance* (the degree of control exerted by a stimulus) (Warriner et al., 2013). Hence, in the case of strongly valenced words it provides a gradient of the diversity of the emotional content within a corpus.

**Figure 3:**
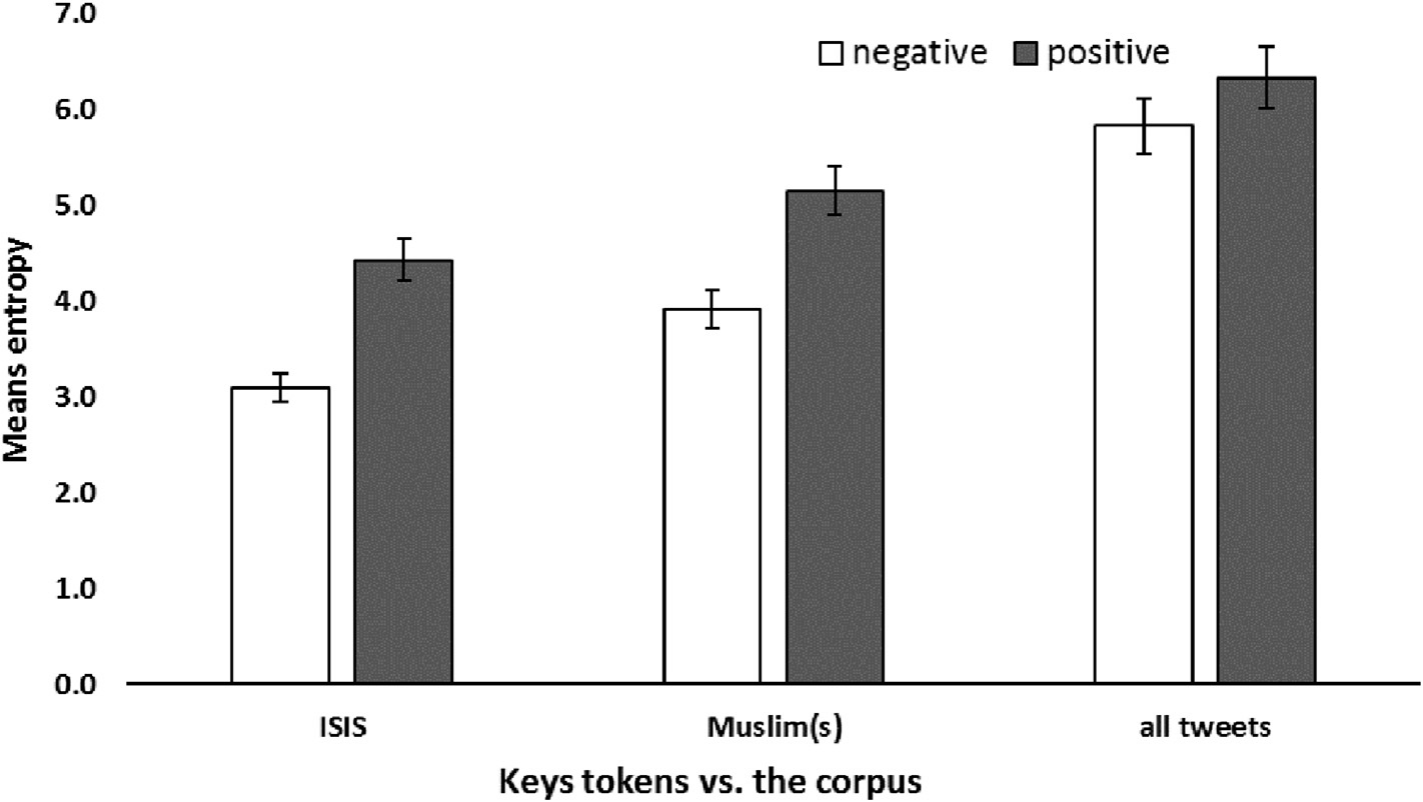
Entropy of positively and negatively valenced words that co-occur with “ISIS”, “Muslim(s)”, and in the corpus as a whole.

For each of the key words, we calculated entropy on the frequency distribution of the 25% most extremely positively and negatively valenced words that co-occur with “ISIS” and “Muslim(s)”, and compared it with extremely valenced words in the corpus as a whole. Results appear in Figure 3. As a rule, entropy is higher for positively than for negatively valenced words. Higher entropy reflects more diversity in the set of positively valenced words co-occurring with “Muslim(s)” in the network, whereas a lower value of entropy for negatively valenced words reflects less diversity in the co-occurring words.

The decrease in lexical diversity based on entropy over time in a closed group has been interpreted as an indirect measure of mutual influence among tweeters over time (Feldman et al., 2018) as well as an indirect measure of tendencies for mutual self-disclosure (Barach et al., 2018). Similarly, Shaikh et al. (2017) reported that it provided a measure of shared knowledge such that lexical diversity increased with increasing distance (measured by geolocation) of the tweeter from the crisis event. As pertains to our data, we interpret decreasing entropy as an indicator of shared or perhaps stereotypic reactions, opinions, and attitudes whereas increasing diversity may be an indicator of an increase in original contributions so as to challenge the emerging collective negative stereotype (See Figure 3).

An analysis of the entropy of the strongly valenced words showed that for positively valenced words that co-occur with the word Muslim(s) the entropy value is 5.1 compared to negative co-occurrence entropy whose value is 3.9. A similar pattern is evident for the ISIS term. Both show reduced lexical variation than for tweets in general. More interestingly, all show lower entropy for negative than for positively valenced terms (see Figure 3).

### 3.3 Differences among tweets based on retweet rate

#### 3.3.1 Word-cloud analysis

One distinct difference among tweets is their distribution or retweet rate. We classified tweets based on those that are retweeted most often (top 2%) and the remainder. We defined tweets that circulate the most frequently as supertweets (*N* = 173; Shaikh et al., 2016) and contrasted the composition of those tweets with that of the remaining tweets (*N* = 55954). The decision of splitting the corpus was motivated by the examination of tweeting rates that exhibit different constellations of either weak or strong users in terms of impactful influence within the studied network. We have considered the constellations around strong influential users (i.e., supertweets) to be more likely produced by active users whereas the remainder of the network is more likely to be produced by passive users. Therefore, supertweets can be defined as the tweets that circulate the most frequently; in other words, they are the most retweeted tweets.

**Figure 4:**
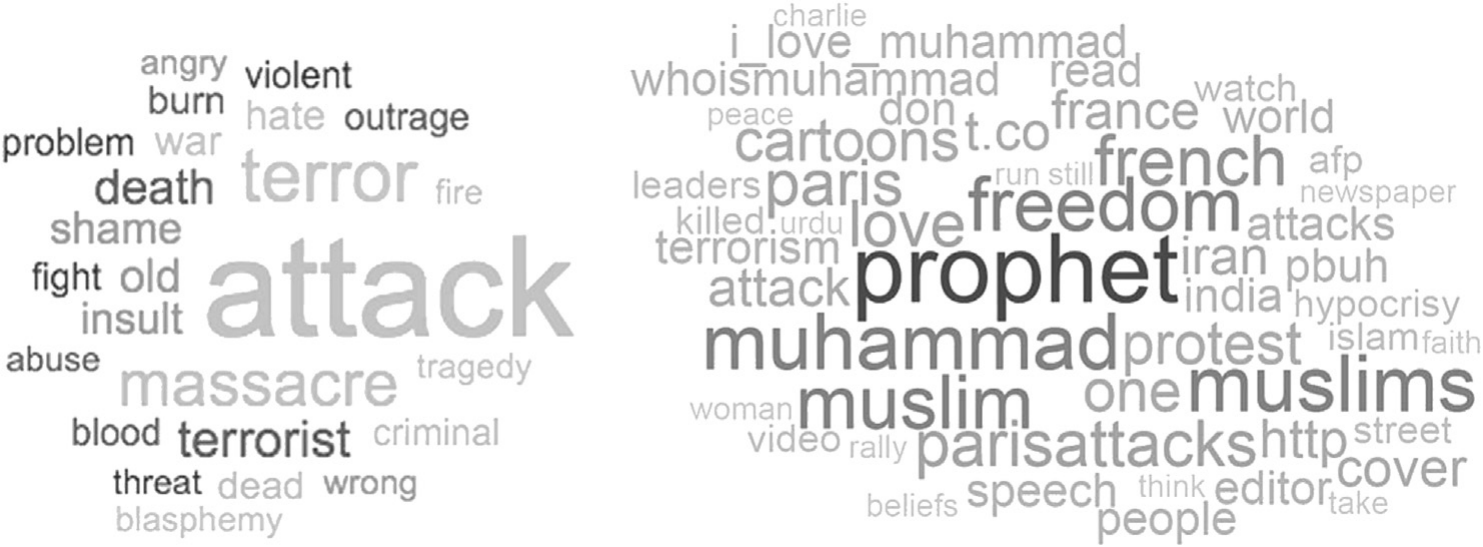
Most common words in the full tweet collection (left panel) and most common words in the super tweet set (right panel).

Figure 4 illustrates the preponderance of negatively valenced words in the cloud for the ***full tweet collection*** (left panel) and the presence of both positively and negatively valenced words in the super tweet corpus (right panel). Word size reflects proportion within the corpus. The results show that the full collection of tweets exhibits almost exclusively negative words. However, the supertweet collection exhibits a very different pattern where the most frequent words include many attributes of Islam such as “Muhammad”, “Muslim(s)”, “prophet”, “I-love-Muhammad”, “who-is-Muhammad”, and positive attributes such as “love” and “peace”. It is also interesting to mention that “ISIS” does not appear in the most frequent words in either of the two word clouds.

#### 3.3.2 Comparison between super-tweets and the non-super tweets

An analysis of the corpus of all tweets including the keywords Muslim and Muhammed produced by the super-tweets (*N* = 173) revealed that the proportion of negatively valenced words (0.4%) coming from super-tweeters is very small compared to the proportion of positively valenced words in tweets (7.1%). A general analysis of all tweets in the corpus of all the tweets coming from the super-tweets (*N* = 173) revealed that the proportion of negatively valenced words in tweets (0.4%) coming from super-tweeters is very small compared to their positively valenced words in tweets (7.1%) towards Muslim and Muhammed in general. An interesting interaction is summarized in Table 2, where the proportions of tweets with extremely negative words in conjunction with Muslims/Muhammed is about the same between the super-tweeters and the remainder of the tweeters (i.e., comparison set) its magnitude is rather small (8%). Of note is that the super-tweet set exhibits a significantly larger proportion of positively valenced words in conjunction with Muslims/Muhammed compared to the remainder set (42% vs. 8%; Z = 16.5, *p* < .0001). This interaction is mirrored in the effect size of the difference between both the number of followers and the number of retweets based on positive content towards Muslim/Muhammed (r = .32 and r = . 60 respectively) versus negative content (r = - .04 and r = - .01 respectively, see Table 2). This result is consistent with a core of super tweeters interested in curbing harsh negative attitudes relative to the whole network/community.

**Table 2:** Comparison of supertweets (N = 173) and tweets sets (N = 55954). with less extreme rates of participation (non-super tweeters) on proportion of key term mentions, the number of followers, and the number of retweets.

	SUPER TWEET SET	COMPARISON SET	
	**Proportions**		**z-test for proportions and r effect size**
Positive towards Muslims/Muhammed	42%	8%	z = 16.5, p < .0001 (r = .24)
Hypocrisy	21%	8%	z = 6.2, p < .0001 (r = .09)
Solidarity	7%	6%	z = 0.5, p = .302 (r = .01)
Negative towards Muslims/Muhammed	8%	8%	z = 0.6, p = .475 (r = .01)

	**Average number of times retweeted**		**Comparisons**
Positive towards Muslims/Muhammed	46%	4%	z = 29.0, p < .0001 (r = .60)
Hypocrisy	25%	3%	z = 16.6, p < .0001 (r = .40)
Solidarity	7%	3%	z = 3.0, p < .005 (r = .07)
Negative towards Muslims/Muhammed	6%	7%	Z = - 0.6, p = .265 (r = - .01)

### 3.4 Social network analysis of co-occurrences over phases

#### 3.4.1 Structure and description of the network graphs

**Figure 5:**
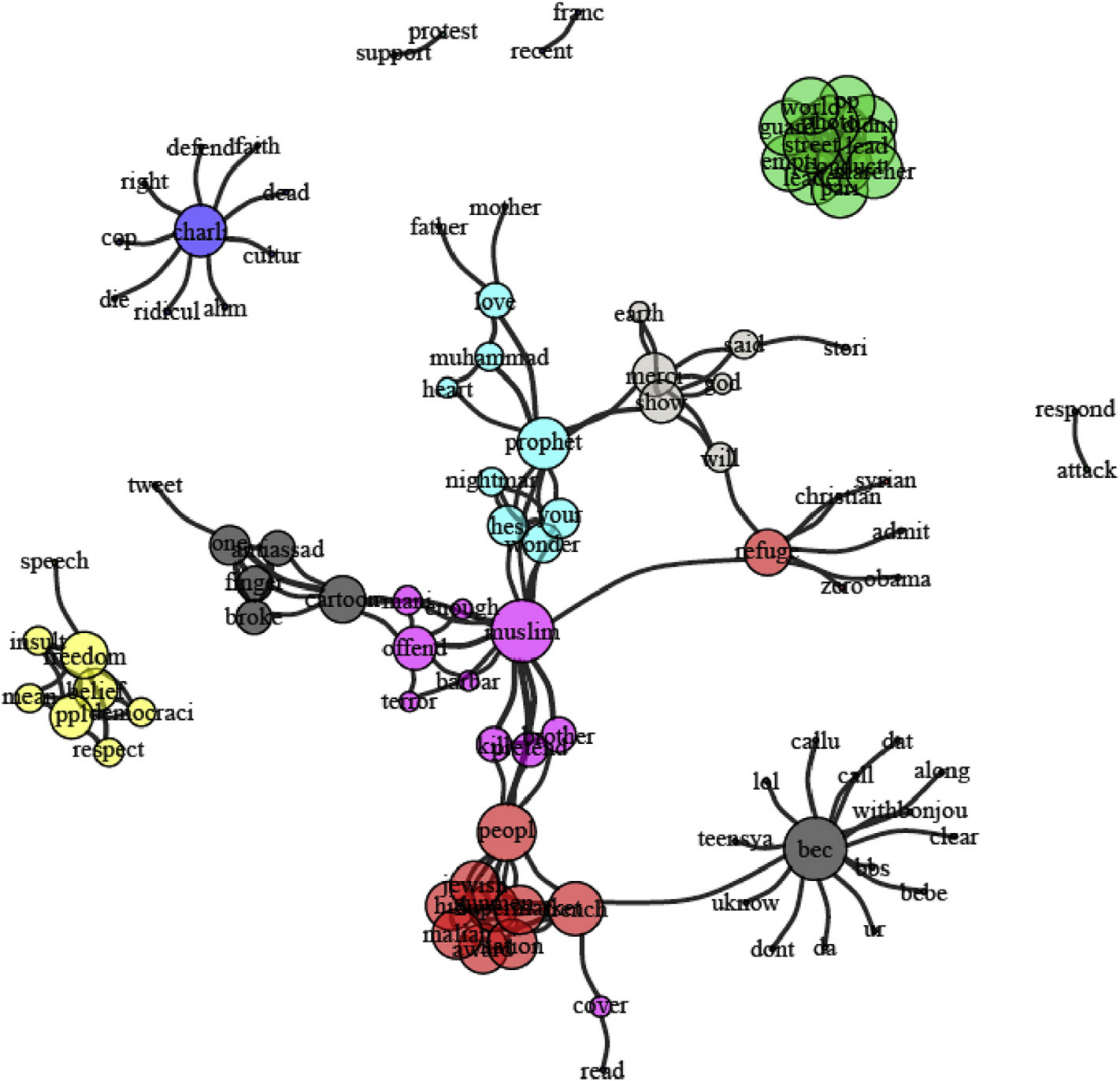
Network graph of the co-occurrences between the supertweets in phase 1. Each community is identified by different color.

**Figure 6:**
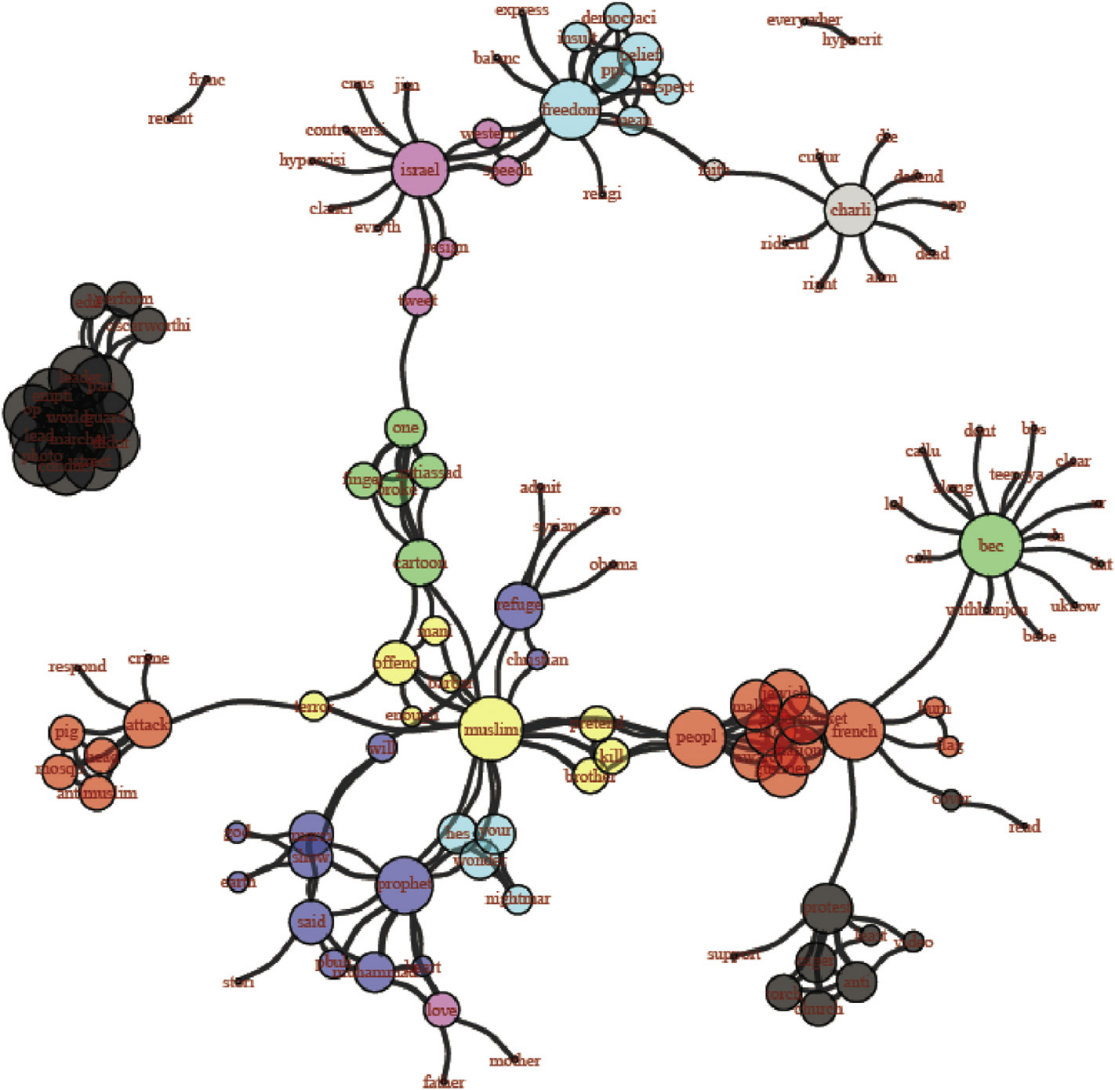
Network graph of the co-occurrences between the supertweets in phase 2. Each community is identified by a different color.

**Figure 7:**
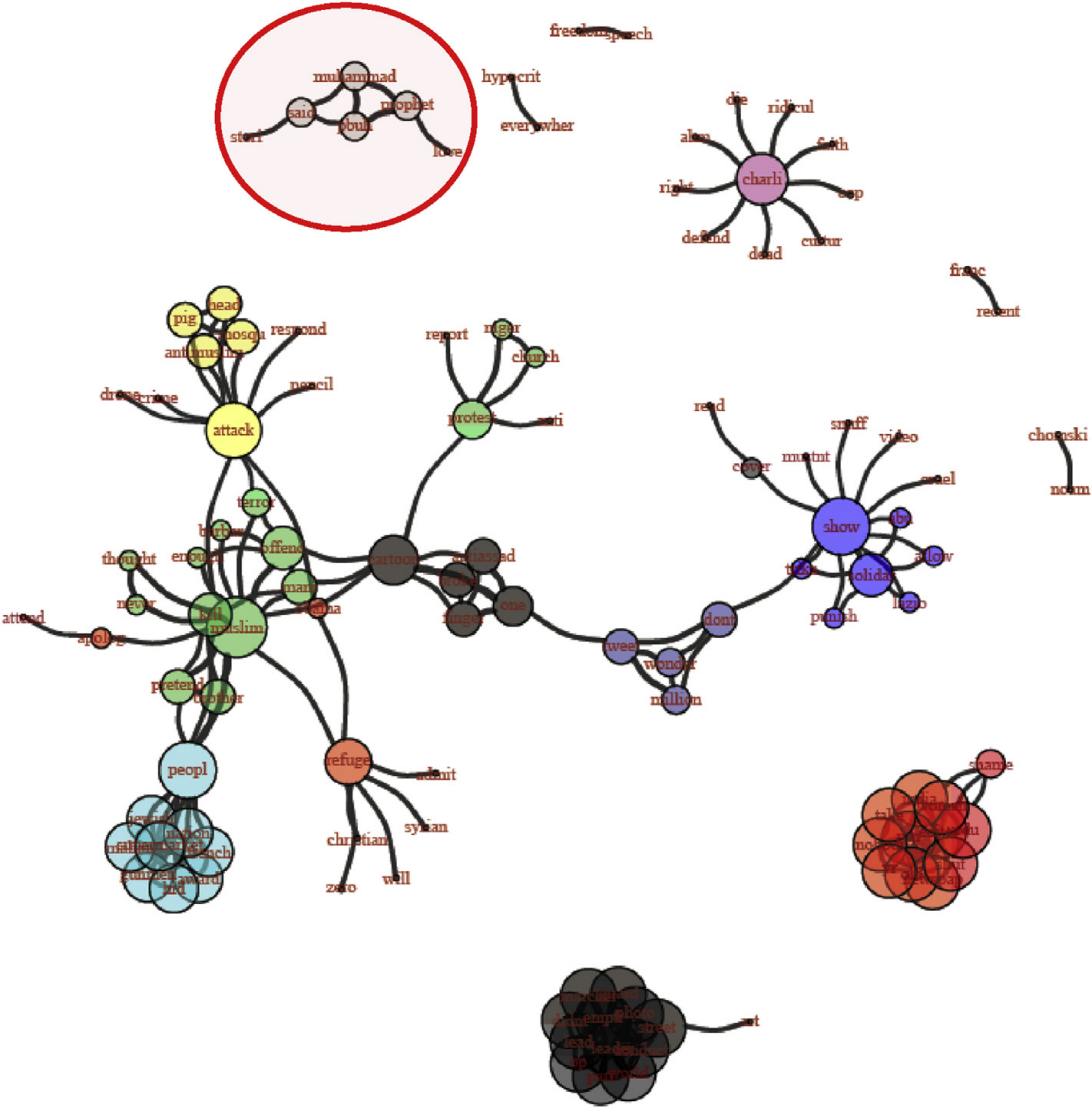
Network graph of the co-occurrences between the supertweets in phase 3. Each community is identified by different color.

For the following analysis, we have pooled tweets and supertweets but have divided the period during which tweets were collected into three phases with each phase corresponding to one third of the data. Dividing the period into three phases (as conducted previously by Shaikh et al., 2016) was a data-driven approach based on an appropriate data binning to ensure equivalent distribution of observations during a period of interest starting from 28-1–2015 until 9-3-2015. We examined words that co-occur together (for at least four times) and compared them in each of the three phases. The network graphs^1^ in Figures 5, 6, and 7 track how these co-occurrence patterns evolve over phases. The nod size in each graph is proportional to each word's frequency in the corpus and edge thickness indicates the co-occurrence strength between two words. We distinguish each sub-community of words by introducing a different color. The techniques of community detection help to find clusters in the network characterized by high density of within-clusters connections and low density of between-clusters connections. The algorithm we used for community detection is Infomap that is known to perform accurately in delivering the correct number of communities of small networks (*N* ≤ 1000) (see Yang et al., 2016 for a review). With Infomap, communities were detected by minimizing the expected description length of a random walk trajectory (Rosvall and Bergstrom, 2008). Lancichinetti and Fortunato (2009) applied many benchmarks in order to compare community detection algorithms and methods. Their results showed that Louvain Community Detection and Infomap have the best performance. In a more recent test of these two methods, Junker (2020) have concluded that Infomap largely outperforms Louvain as long as they were not applied on large networks, which is exactly the scenario of the current study.

##### 3.4.1.1 Phase 1

The graph for phase 1 in Figure 5 revealed seven main communities containing a total of 100 vertices that were grouped according to their proximity in the corpus (see also Table 3). In phase 1, lexical elaboration of key terms is not salient; we can detect a few elements around the “prophet” persona in turquoise that have started to emerge but none is well enough elaborated to form a stand-alone cluster.

**Table 3:** Comparison centrality measures in each phase.

	Network 1 (*phase 1*)	Network 2 (*phase 2*)	Network 3 (*phase 3*)
Average Degree	4.38	4.32	4.80
Average Closeness	0.00	0.00	0.00
Average Betweenness	57.60	224.12	65.57
Edge density	0.044	0.033	0.042
Transitivity	0.72	0.64	0.80
Coreness	3.55	3.39	4.15
Number of communities	7	4	9

##### 3.4.1.2 Phase 2

In the second phase, the number of significant communities or clusters has diminished to four instead of the seven in phase 1. Note that almost all of the elements related to the Islam world have started to cohere and the “Prophet” theme in violet has started to emerge as a separate cluster with various other connections incorporated with it (see Figure 6).

##### 3.4.1.3 Phase 3

In phase 3, the “prophet” theme in the red circle is now a well-defined and autonomous cluster among the nine communities that compose the network of co-occurrences in this phase of the corpus (see Figure 7). More prominent than the pattern of lexical diversity and valence that we reported with the entropy measure for all tweets - and consistent with the contrast between super-tweets and the comparison group - in phase three, key terms undergo a significant change in the narrative reflected by more positively valenced words as shown above.

#### 3.4.2 Centrality measures analyses of the network graphs

Centrality measures are designed to determine the most important or central element, also referred to as vertex or node, in this network. Many indices have been developed. Table 3 shows the major centrality measures to compare the three networks as function by their phase.

**Figure 8:**
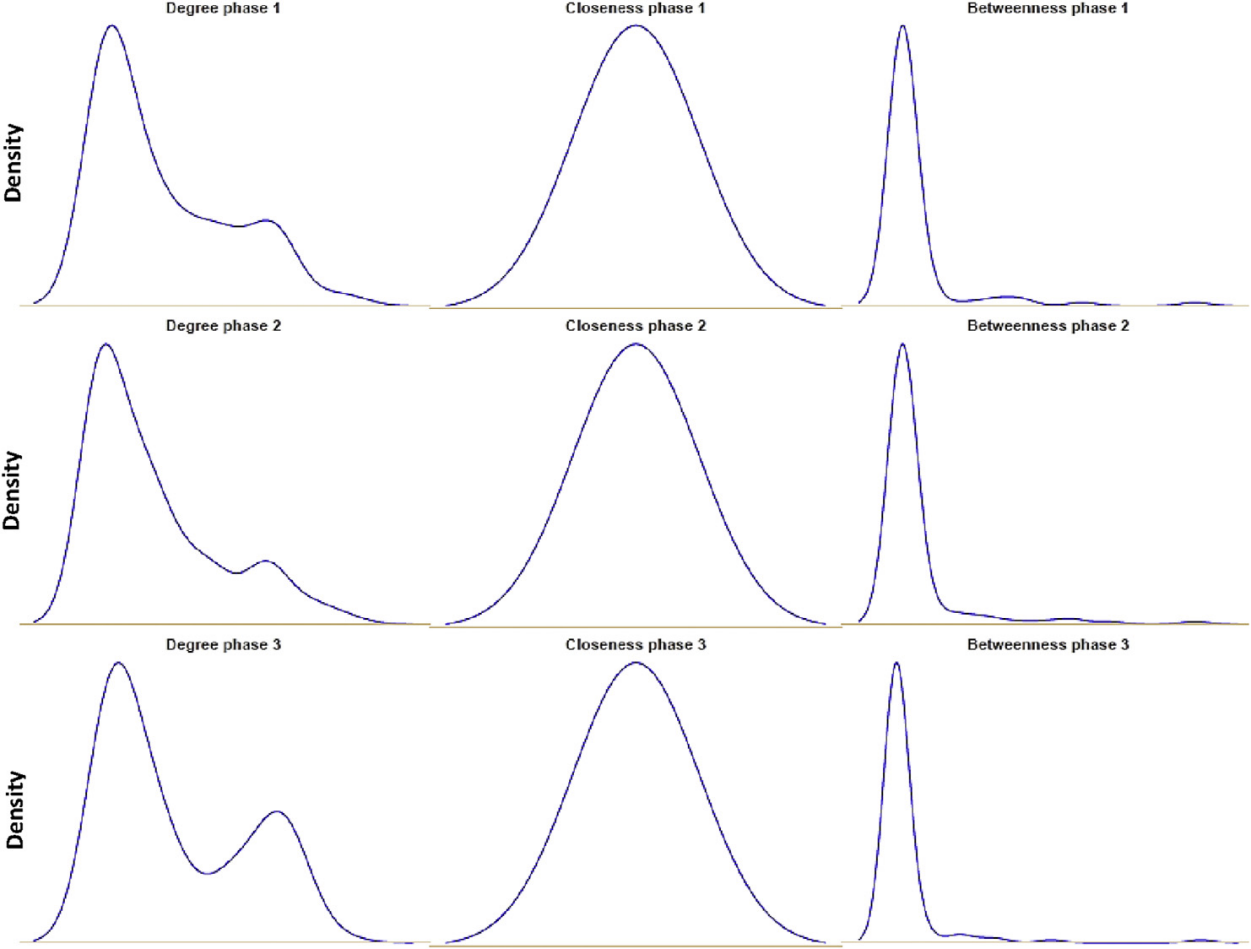
Distribution plots of the Degree (left panels), Closeness (middle panels), and Betweenness (right panels) per network/phase.

A visualization of the density for Degree, Closeness, and Betweenness centralities in Figure 8 clearly shows that the major differences between phase 3 and the 2 previous ones lie on the presence of a second peak rendering a bimodal-shaped distribution for the Degree centrality measure. This is more likely to capture the emergence of a second behavior in the network. Similarly, for the Betweenness centrality measure - that reflects the number of shortest paths that pass through each vertex - the distribution for phase 3 exhibits a more pronounced leptokurtic shaped curve compared to Phase one and Phase two. This is characteristic of the presence of extreme values for some key terms in the network (see Figure 5).

For the three networks, the edge density score – that reflects the portion of the potential connections between “nodes”– are similar which indicates that all three networks are rather sparse graphs.

For the transitivity measure, network 3 again deviated by showing the highest probability (0.8). This means that, on average, the chance that two terms (nodes) that share a common word co-occur together is almost half. This is a rather high probability compared to network one and two (0.72 and 0.64 respectively). The fluctuation of this measure captures the dynamics of the network over time since it indicates that terms tend to occur in distinct contexts and that participants use individually the same terms in similar contexts.

Coreness is a useful index to understand how clustering operates within elements of a network and to track the emergence of communities (i.e., Seidman, 1983). The k-core of a graph corresponds to the maximal connected subgraph whose vertices are at least of degree k within the subgraph. In other words, a k-core is an area of a high cohesion between elements within the network (Scott, 2013).

**Figure 9:**
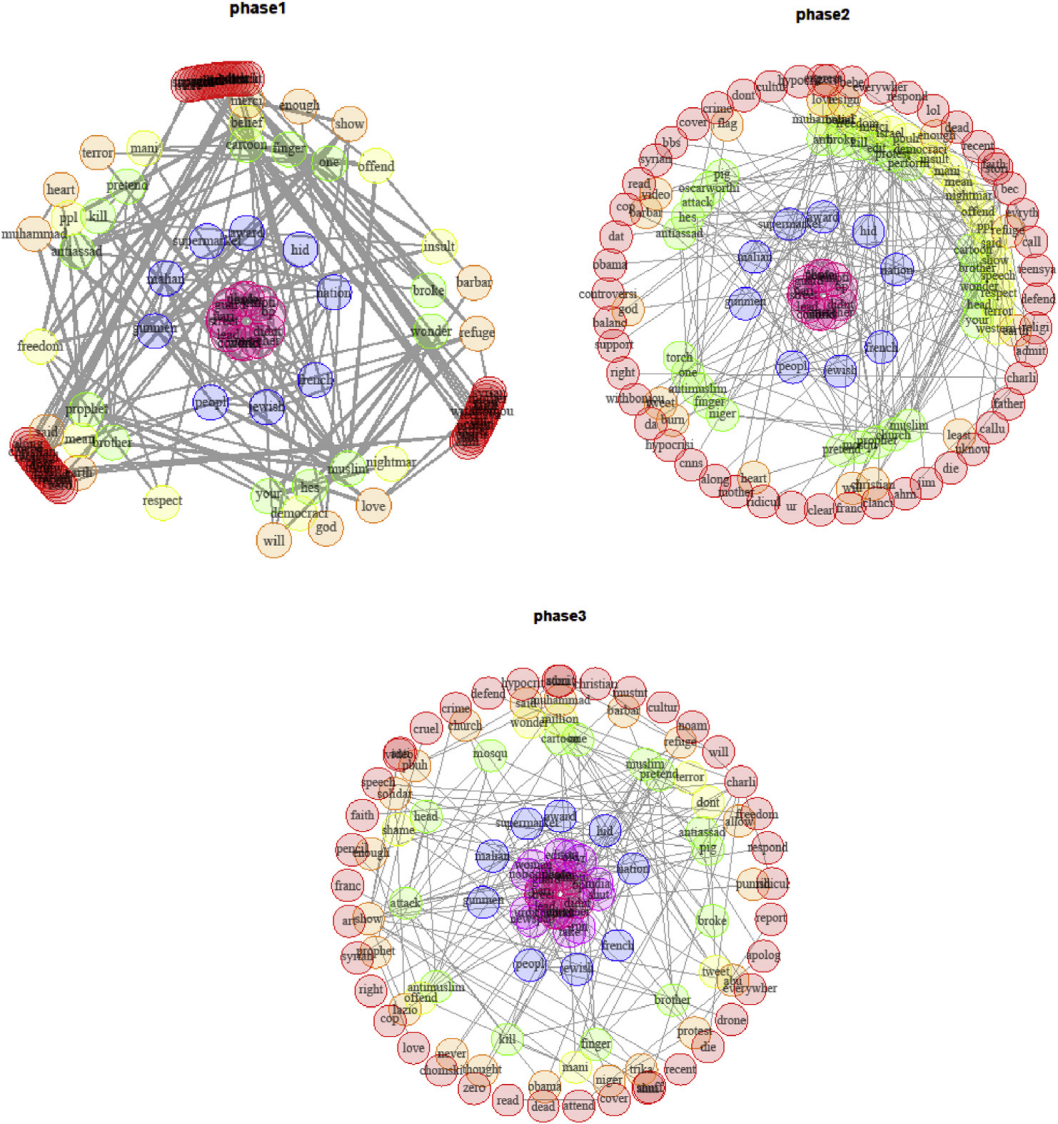
K-core decomposition of the three networks from phase 1 to phase 3 of the co-occurrences in the supertweets corpus. Lower k-shells contain words (nodes) at the periphery of the network; higher k-shells contain core nodes. Each node color matches a cluster. Five distinguished shells formed the structure of the coreness in the final graph for phase 3.

Finally, we apply the K-core decomposition technique to each of the three networks in order to determine the core-periphery relationships in each of the three phases. Results are depicted in Figure 9. Terms related to the incidents (e.g.: “supermarket”, ”Jewish”, “hid”, “gunmen”) of Charlie Hebdo shooting and the attack that occurred at a kosher supermarket two days later where the gunmen were arrested) occupy the core blue layer of each network. The flow from the core to the periphery shows progressively more structure as one moves from network (phase) 1 to network (phase) 3. In the latter, the shells form a clear structure of coreness.

Finally, we have applied on the supertweets corpus a very recent approach that combines the empirical estimate of Shannon entropy and the species accumulation curve (Chao et al., 2013) in the three phases in order to capture dynamic aspects of our population diversity (e.g., Deng et al., 2015) and by extension the intensity of the consensus. The values shown in Table 4 were obtained via bootstrap method and the results revealed that phase 3 has the lowest entropy/or lexical diversity as compared with phase 1 and phase 2, which is in line with our hypotheses that the network is evolving towards a global consensus as shown in the previous results sections.

**Table 4:** Estimation of transformed Shannon diversity based on the method proposed by Chao et al. (2013) and the associated confidence interval in the three phases.

	Observed	Estimator	95% Lower	95% Upper
phase1	3.636	3.637	3.636	3.65
phase2	4.204	4.206	4.204	4.217
phase3	3.115	3.117	3.115	3.132

## 4 Discussion

The goal of the present study was to examine the evolution of knowledge, beliefs, and expectancies in naturally occurring social network based on the content of tweets. We tracked positively and negatively valenced words elicited on Twitter in response to a traumatic event collectively experienced at varying degrees of immediacy. We further demonstrated how a micro-blogging system such as Twitter can provide a practical platform within which people share latent opinions and beliefs as well as more overt reactions as shown in some previous studies (e.g., Del Vicario et al., 2017; Goldstone and Lupyan, 2016; Stadthagen-González et al., 2018). In the case of the Charlie Hebdo shooting, the hashtags #JeSuisCharlie and #JeNeSuisPasCharlie became the anchor of many positive and negative reactions. By revisiting the corpus collected by Shaikh et al. (2016), we were able to look at the data for evidence to support the hypothesis that a backlash effect arose to offset harsh negative attitudes that stemmed from stereotypes about a particular social group.

Our analysis entailed contrasting two subsets of tweets, one associated with the super-tweeters characterized by very high participation rates and another with the remainder of the contributors. Comparisons based on entropy analyses showed decreasing lexical diversity, indicating many words used and reused by users and this was depicted in the network graphs. We interpret the outcome as an indicator of the convergence of opinions and attitudes and reactions and link them to stereotypes. Conversely, the set of supertweets exhibited progressively more diversity and creativity, which we interpret as an indicator of non-conformity to a collectively shared negative attitude within the network as a whole. Our findings along with previous attempts to understand sentiment data from Twitter (Fischer and Reuber, 2011; Giglietto and Lee, 2017; Shaikh et al., 2016) show that the lexical diversity in conjunction with the constraints on message length imposed by Twitter can be highly informative about community intentions, opinions, and contradictions.

The second key finding pertains to the dynamics of the supertweeters and their reactions over time as captured in the similarity analysis that was performed at each of three phases. The results revealed that in phase 1, few elements were detected around the “Prophet” persona. In phase 2, the Prophet theme has started to emerge as a separate cluster after incorporating various connections from the earlier Charlie Hebdo theme, but in phase 3, the prophet cluster significantly disconnects from many other key nodes of the Charlie Hebdo theme. For example, “insult/offense” and “hypocrisy” were close to 5% in the early period but dropped to 1% or lower at the end of the third period of observation (See Table 2).

A tentative explanation of the general pattern that we describe can be drawn from the Rudman and Fairchild (2004) model on the role of backlash in stereotype maintenance. The authors distinguish between actors and perceivers of cultural stereotypes based on differing roles, involvement timeline, and reactions. In our database of online communication, it is not meaningful to tag each user as exclusively an actor or a perceiver since in social media both roles can be assumed to be easily interchangeable. Instead, the classification is based on degree of participation conceptualized as gradation of engagement rather than as discontinuous roles. The underlying logic is that those who adhere more to the status quo and thus retain cultural stereotypes function as the online variant of perceivers, whereas those who are more engaged (e.g., actors) and serve to offset the status quo function as the online variant of actors and backlash evolves as their recovery strategy. In the context of Charlie Hebdo, super-tweeters gradually demonstrated an opposite and compensatory reaction relative to the collective expectations of the whole and that reaction constituted a violation of the negative social expectations. A preponderance of strongly negative words is consistent with an anti-Muslim stance whereas that of strongly positive words depicts a backlash effect whose function is likely to alter the narrative so as to tone down the intensity of stereotyping associated with Islamophobia.

More specifically, the supertweeter stream continuously fed the network with justifications and arguments, many of which evoked the “prophet” persona and these served to offset many negative stereotypes toward the Muslim community. Above we depicted many analyses in which the prophet persona evolved from phase 1 to phase2. Here examples of tweets from the corpus in phase 2: *“#I_love_Muhammad my prophet said: "Show Mercy to those on earth, and God will show Mercy to you*” and in phase 3: “*Still trending is #WhoIsMuhammad - a response to the latest #CharlieHebdo cover*”. This pattern is partly corroborated in a recent study by Nakayama et al. (2019) where a small group of participants repeatedly performed a cognitive task. The authors allowed the participants to change their answers based on the answers of other participants in each round of the cognitive task. In their context, the adaptive pattern of how inter-individual relationships spontaneously emerged showed the evolution of a small network of people that was driven by correct responses. In our findings, the emergence of strong links overtime was triggered by more global attitudes rather than responses in a simple laboratory task and the role of positive feedback between opinions and sentiments was necessarily restricted to a much smaller subgroup. Stated alternatively, the small world of the supertweeters in our data exhibited progressively more positively valenced tweets about particular aspects of Islam that were unlike those of the remainder of the network.

The peripheral community formed around the persona of the Prophet of Islam was depicted in the co-occurrences between terms shown in phase 3 and provides a second perspective on the pivotal role played by the “critical” sub-community of supertweeters who through their tweeting behavior succeeded in changing the sentiment of the whole network from negative to positive. The impact of supertweeters is in line with the insights of Barberá et al. study (2015) who reported that minorities often constitute the locus of changes in a large-scale network not because of their number but because of their commitment and their aggregated contribution to the spread of consistent protest messages. In Ch'ng study's terms, a sub-community can maintain the boundaries and cohesiveness of a community within a larger network by adhering to arguments with a shared content (Ch'ng, 2015). Ultimately, their content conveyed a counter-stereotypical message in line with universal and highly socially desirable values pertaining to peace and love to all human kind. Similarly, the CharlieHebdo sub-community of supertweeters established their coherence around the primary teachings of the Prophet of Islam. Note that this explanation of the reaction is linked to exposure to the media's biased framing of the “Je Suis Charlie” incident. A media portrayal with reference to western transgressions and abuses with less attribution of responsibility for the attack to Islam would have triggered a very different community of supertweeers than framing the incident in terms of American victimization (9/11 attack). Only the latter implicates the perceived culpability of Islam (see Walter et al., 2016).

The late emergence of rational and less impulsive discourse associated with the prophet theme mentioned above can be contrasted with the use of strong emotional reactions at the advent of the event. Emotionality in the content of the tweets also differentiates the supertweeters as a small-world network from the remainder of users in the network as a whole (Bodrunova et al., 2018; Ch'ng, 2015; Del Vicario et al., 2017). Here again, our results confirm an evolution in the core reverberation from the beginning of the incident.

Finally, we attempted to match emotionality with the inclusion of emoticons in the tweets from our network. The results showed that the peak of emoticon use was on January 15, 2015 (*N* = 40176, 7.62% of the total of tweets) whereas in the last day of our data collection only 2048 (0.38% of) tweets included emoticons. This pattern suggests a parallel between attention and emotion in that Fusaroli et al. (2015) reported that after an initial burst, public attention slowly decays through the course of the event.

It is worth noting that analyses of aspects of behavior covering political and social uprisings, online video gaming, rumor spreading, internet memes contagion as well as online collective behaviors more generally can be framed within a model of Virtual Collective Consciousness (VCC) that emerges from the homogeneity, the synchronicity, and the spontaneity of users (Marzouki et al., 2012, 2014; Marzouki and Oullier, 2015). The emergence and the thriving of a collective consciousness stems from complex interactions among users and asymptotes when a consensus can be reached and individual online behaviors coalesce towards a common goal. VCC invites comparison with agent-based models where a system of regularities emerges from local interactions between multiple agents each of which sees their own rules and states changing through experience and interaction (Skvoretz, 2002). This framework can be applied to our data because individual users within a small-world network act according to their own rules in tweeting positively about the Prophet of Islam while using network reactions and feedback within the network to learn how to better shape the content of their tweets to meet a common goal. As for the small group of supertweeters, the phenomenological experience of “feeling togetherness online” can foster and enhance that cohesiveness and synchronicity (Osler, 2019). Indeed, the sharing of emotional experience as reflected by the “we-experience” (e.g., Zahavi, 2015) has been characterized by common intentionality, reciprocal awareness, interdependency, and affective requirement (Osler, 2019). Indeed, overtime, the clusters we targeted became less central and assumed greater distinctiveness. Early on, the “Muslim” cluster separates from the “People” and “Prophet” clusters. Then in phase 3, the newly formed stand-alone cluster around “Prophet” became progressively more elaborated by joining with “Muhammed” and other terms such as “said” in references to his sayings taken as a source to promote peace and “love”. The latter is another term joining the cluster.

In closing, our findings extended to the case of tweeting behavior within online small-world networks, the conclusion of a recent paper by Peters et al. (2017) who asserted that deviant social behaviors, in our case terrorist attacks, serve to draw people's attention to existing social norms and, ultimately, to either challenge or endorse those norms. Their insight from the study of online behaviors within sub communities is that coming in contact with “deviant behaviors” may trigger many novel forms of social functioning, as they bring into focus ideas and beliefs that help us understand and clarify some of the underlying norms.

## Declarations

### Author contribution statement

Y. Marzouki: Conceived and designed the experiments; Analyzed and interpreted the data; Contributed reagents, materials, analysis tools or data; Wrote the paper.

S. Shaikh: Conceived and designed the experiments; Performed the experiments; Analyzed and interpreted the data; Contributed reagents, materials, analysis tools or data.

L. B Feldman: Conceived and designed the experiments; Analyzed and interpreted the data; Wrote the paper.

E. Barach: Performed the experiments; Contributed reagents, materials, analysis tools or data.

V. Srinivasan: Contributed reagents, materials, analysis tools or data.

### Funding statement

This research did not receive any specific grant from funding agencies in the public, commercial, or not-for-profit sectors.

### Competing interest statement

The authors declare no conflict of interest.

### Additional information

No additional information is available for this paper.

## Footnotes

1 Figures 5, 6, 7, and 9 can be viewed and downloaded in high resolution via this link: https://osf.io/5czf8.
